# WHO EXPERIENCES MASS MARKETING FRAUD IN AMERICA? A SURVEY OF KNOWN VICTIMS

**DOI:** 10.1093/geroni/igae098.1398

**Published:** 2024-12-31

**Authors:** Marguerite DeLiema, Siyu Gao, M Daniel Brannock, Lynn Langton

**Affiliations:** University of Minnesota Twin Cities, St. Paul, Minnesota, United States; University of Minnesota, Minneapolis, Minnesota, United States; RTI International, Research Triangle Park, North Carolina, United States; RTI International, Research Triangle Park, North Carolina, United States

## Abstract

Significant research has attempted to understand the characteristics of older adult fraud victims, yet most surveys rely on participants’ self-reports of victimization. This approach can lead to biased estimates because older victims are less likely to disclose victimization experiences relative to younger victims. We avoid underreporting bias by surveying a population of victims identified by the US Postal Inspection Service. All respondents mailed money in response to fraudulent solicitations between 2022 and 2023 (range = 1 to 168 victimization incidents). Victims’ payment envelopes were detained by Postal Inspectors as part of the agency’s law enforcement efforts. Victim addresses were securely shared with the research team. The survey response rate was 23% (N=934); 84% of participants responded by mail and 16% responded online. Average age was 74 (standard deviation = 13.2; range= 20 to 98). Mass marketing mail fraud victims were racially and economically diverse: 22% of victims were Black, 10% were Latino/a, and 6% were Asian or Pacific Islander. A quarter reported an annual household income of less than $20,000; 27% reported a household income between $20,000 and $50,000. Rates of widowhood and divorce were high—31% and 20%, respectively. Nearly half of fraud victims reported that they live alone. These results suggest that diverse, lower income and isolated older adults are targeted by mass marketing fraud. Given the relatively robust response rate, consumer fraud education materials should be mailed to victim households and include information on how to identify fake solicitations and report scams.

